# Poking Fun

**Published:** 1871-11

**Authors:** 


### POKING FUN.

An Alabama editor, having read our book on "Sleep, or the Hygiene of the Night," says, in reference to certain suggestions there, "Dr. Hall's book advises that husband and wife should sleep in different apartments; Dr. Hall can sleep where he chooses, but for himself he intends to sleep where he can defend his wife against rats and other nocturnal foes, as long as he has one to defend."

A gallant gentleman and a cavalier of the old school is this same Alabama editor. As he is good-natured we would like to argue the case with him. Let him try sleeping in a large, light, and airy room, fifteen feet long and broad, on a fine hair mattressed bed, broad enough for three, and let his wife and the baby sleep in a similar room, communicating though, for easy call, in case of the intrusion of burglars, rats, and other things, and see if great advantages will not result from it.

The book mentioned shows the necessity of sound, connected sleep in large, well ventilated rooms, for all, especially for men engaged in business, whose mental faculties are largely used. Suppose there is a baby in the case; it is sure to be wriggling and twisting and crawling around, about daylight, just at the time that sleep is felt to be the most delicious, exploring the nostrils with the finger, gouging out the eyes, pulling the hair, prying into the mouth to see what is down there; every now and then breaking out into an infantile crow, thereby knocking the snooze into a teetotally cocked hat, and ruthlessly breaking into other arrangements. If there can be any good sleep, any enjoyable health, vigor, and joyousness in any such proceedings as these, then we are greatly mistaken.

If two are in the same bed, every time one turns over during the night, or plunges into one another with fist or feet, there is a rousing up, with danger of some wrathfulness, and more or less difficulty in getting to sleep again; the only way to prevent such untoward circumstances, if our Alabama friend and his wife will sleep in one and the same bed, is for each to get as far apart as possible, get to the farthest edge asunder, and sleep on the bed rail, leaving all the best part of the bed unoccupied. How long it would remain unoccupied let any fair minded person judge. We pause for a reply.

Besides showing the importance of sleeping in a healthful atmosphere, as we pass one third of our existence in our chambers, the book goes on in detail to lay down rules and regulations, times and seasons, for what is connected with our sleeping habits. It is a book which young and old, married and unmarried could read with great profit, as it is specific and practical on all matters connected with the general subject. Not only is it valuable to old men, but to youth, especially as it communicates most important information in reference to a class of subjects on which much has been written by evil minded and unprincipled men, whose deliberate object is to mislead, to alarm, to corrupt, and then to fleece. We wrote the book with many misgivings and questionings as to its propriety and necessity; but there was so much that was mischievous, untruthful, and demoralizing in publications which flooded the country, that the inquiry was excited, Shall poison be spread broadcast, and no antidote whatever be provided? It is a dollar and a half book, and is very suggestive on all subjects treated.

---

Coughs, Colds, and Consumptions are best avoided in winter by dressing abundantly warm, keeping the feet dry and comfortable, and never remaining long enough in a still position to allow a feeling of chilliness to run over the system.

